# Synthesis, carbonic anhydrase inhibition studies and modelling investigations of phthalimide–hydantoin hybrids

**DOI:** 10.1080/14756366.2024.2335927

**Published:** 2024-04-12

**Authors:** Morteza Abdoli, Alessandro Bonardi, Paola Gratteri, Claudiu T. Supuran, Raivis Žalubovskis

**Affiliations:** a Institute of Chemistry and Chemical Technology, Faculty of Natural Sciences and Technology, Riga Technical University, Riga, Latvia; b Department NEUROFARBA – Section of Pharmaceutical and Nutraceutical Sciences, Laboratory of Molecular Modeling Cheminformatics & QSAR, University of Florence, Sesto Fiorentino (Florence), Italy; c Department of NEUROFARBA – Section of Pharmaceutical and Nutraceutical Sciences, University of Florence, Sesto Fiorentino (Florence), Italy; d Latvian Institute of Organic Synthesis, Riga, Latvia

**Keywords:** Carbonic anhydrase, inhibitors, hydantoins, phthalimides, docking studies

## Abstract

A novel series of hydantoins incorporating phthalimides has been synthesised by condensation of activated phthalimides with 1-aminohydantoin and investigated for their inhibitory activity against a panel of human (h) carbonic anhydrase (CA, EC 4.2.1.1): the cytosolic isoforms hCA I, hCA II, and hCA VII, secreted isoform hCA VI, and the transmembrane hCA IX, by a stopped-flow CO_2_ hydrase assay. Although all newly developed compounds were totally inactive on hCA I and mainly ineffective towards hCA II, they generally exhibited moderate repressing effects on hCA VI, VII, and IX with *K*_I_s values in the submicromolar to micromolar ranges. The salts **3a** and **3b**, followed by derivative **5**, displayed the best inhibitory activity of all the evaluated compounds and their binding mode was proposed *in silico*. These compounds can also be considered interesting starting points for the development of novel pharmacophores for this class of enzyme inhibitors.

## GRAPHICAL ABSTRACT

**Figure:**
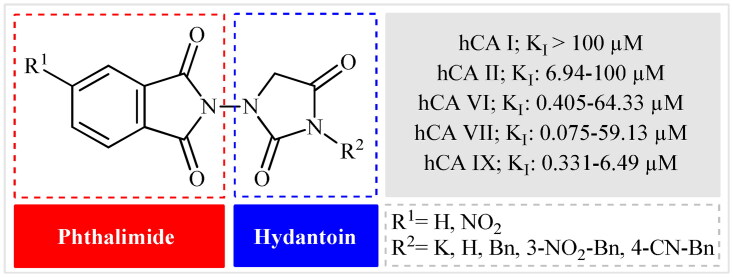

## Introduction

The carbonic anhydrases (CAs, EC 4.2.1.1) are superfamily of metallo-enzymes that are found predominantly in both prokaryotes and eukaryotes and categorised into eight major groups (α, β, γ, δ, ζ, η, θ, and ι) based on their amino acid sequence similarity.^1^ Human (h) CAs belong exclusively to the group of α-CAs and comprise 15 different isoforms (CA I, II, III, VA, VB, VI, VII, VIII, IX, X, XI, XII, XIII, XIV, and XV) ^2^. There is increasing evidence that abnormal levels and/or activities of these enzymes lead to a variety of diseases (e.g. glaucoma, oedema, epilepsy, obesity, sterility, and cancer) ^3^. Therefore, selective inhibition of hCA isozymes is an important approach for discovery and development of safe and effective medicines.^4^

**Figure 1.**
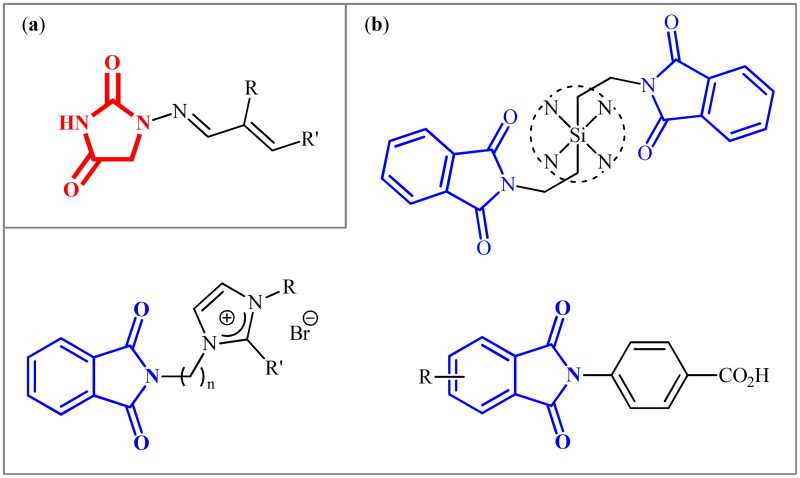
(a) General structure of furagin derivatives developed by our group as isoform-selective CAIs; (b) selected examples of phthalimide-based CAIs.

The hydantoin (or glycolylurea, imidazolidine-2,4-dione) moiety is a *N*-heterocyclic structural scaffold frequently present in various natural products^5^ and synthetic pharmaceuticals^6^ with diverse nature of activities from anti-bacterial to anti-androgen or anticonvulsant action. Similarly, phthalimide (or isoindoline-1,3-dione) is a ubiquitous structural motif in various natural products^7^ and biologically active pharmaceuticals.^8^ Due to their diverse properties, many researchers have been working to explore these 1,2-dicarboxamides to their maximum potential against several diseases or disorders.^9–13^ In this context, recently, we disclosed that the clinically used antibiotic Furagin (Figure 1(a)) and its derivatives, which contain a hydantoin core, display effective inhibitory activity on several human (h) CAs (EC 4.2.1.1)^14^. Several phthalimide-based compounds were developed by various research groups (Figure 1(a)), which showed appreciable activity against various hCA isozymes.^15^

**Figure 2.**
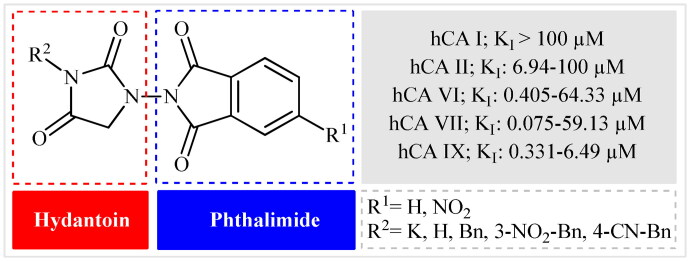
General structure of phthalimide–hydantoin hybrids investigated in the paper.

Keeping all the above facts in mind, and with consideration that the incorporation of different pharmacophores usually leads to the formation of more active compounds,^13^ in connection with our works on the field of drug design of CAs inhibitors, herein, we decided to synthesise a series of hitherto unknown phthalimide–hydantoin hybrids and investigate their inhibitory capability against five hCAs isozymes: the cytosolic isoforms hCA I, II, and VII as well as the secreted isoform hCA VI and trans-membrane tumour-associated isoform hCA IX (Figure 2).

## Results and discussion

### Chemistry

**Scheme 1.**
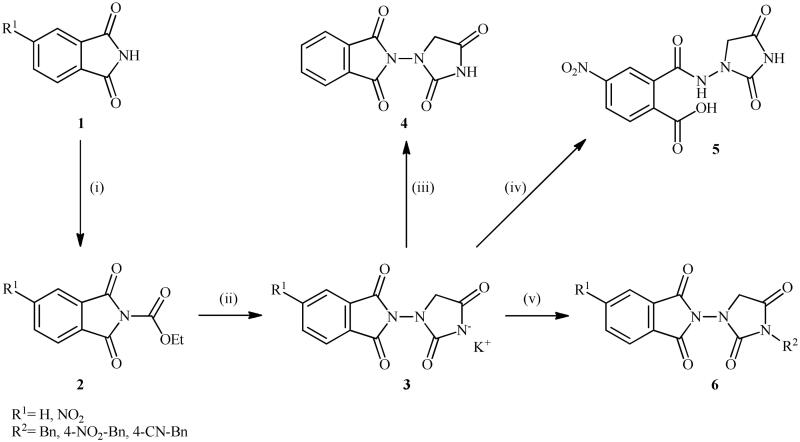
Reagents and conditions: (i) ClCO_2_Et (1 equiv.), 0–20 °C, 4 h, 31%; (ii) 1-aminohydantoin hydrochloride (1 equiv.), K_2_CO_3_ (2 equiv.), 100 °C, 20 h; (iii) H_2_O/HCO_2_H (10:1), 70 °C, 2 h; (iv) H_2_O/HCO_2_H (10:1), 50 °C, 30 min; (v) (a) K_2_CO_3_, DMF, 100 °C, 1.5 h; (b) Bn-Br (1.5 equiv.), DMSO, 100 °C, 5 h.

The synthesis of the target phthalimide–hydantoin hybrids is shown in Scheme 1. The synthesis started from phthalimides **1**, which were converted to the corresponding *N*-ethoxycarbonylphthalimides **2** via treatment with ethyl chloroformate (ClCO_2_Et) in a basic medium. The phthalimide–hydantoin hybrid salts **3** were then prepared through the reaction of appropriate *N*-ethoxycarbonylphthalimide **2** with commercially available 1-aminohydantoin hydrochloride in the presence of 2 equiv. K_2_CO_3_ in NMP at 100 °C. Neutralisation of unsubstituted intermediate **3a** led to the formation of the expected phthalimide–hydantoin hybrid **4**, while in the case of NO_2_-substituted one **3b**, a ring opening took place to give **5** as the sole product. There is no need to say that strongly electron-withdrawing nitro functionality make the carbonyl groups of phthalimide core in compound **3b** more electrophilic and increase their reactivity towards hydrolysis. In parallel, acidic medium may also increase the rate of this ring opening reaction by acting as a catalyst. Finally, potassium 2,5-dioxoimidazolidin-1-ides **3** were reacted with a series of benzyl bromide derivatives in the absence of any additive, leading to the desired *N*-benzyl substituted derivatives **6**.

### Carbonic anhydrase inhibition

All of the newly synthesised compounds (**3–6**) are tested *in vitro* for their inhibitory activities against five hCAs isozymes: the cytosolic isoforms hCA I, II, and VII as well as the trans-membrane tumour-associated isoform hCA IX and secreted isoform hCA VI, by means of the stopped-flow carbon dioxide hydration assay.^16^ Their inhibition profiles are compared to the acetazolamide (AAZ; standard reference inhibitor for hCA) as outlined in Table 1.

**Table 1.** Inhibition data of human CA I, II, VI, VII, and IX with phthalimide–hydantoin hybrids (**3–6**) and the reference drug (AAZ) by a stopped-flow CO_2_ hydrase assay [16].

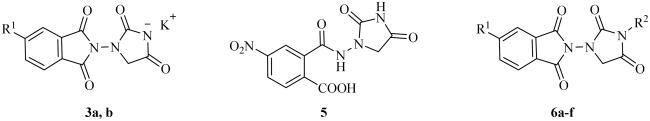							
Cmpd	R^1^	R^2^	*K*_I_ (μM)^a^				
			hCA I	hCA II	hCA VI	hCA VII	hCA IX
**3a**	H	–	>100	6.94	0.405	0.075	0.331
**3b**	NO_2_	–	>100	8.99	0.701	0.163	0.488
**5**	–	–	>100	58.58	10.54	13.88	4.06
**6a**	H	Bn	>100	>100	57.67	33.72	3.73
**6b**	NO_2_	Bn	>100	>100	40.19	45.94	2.73
**6c**	H	4-NO_2_-Bn	>100	>100	50.18	37.63	5.68
**6d**	NO_2_	4-NO_2_-Bn	>100	>100	49.81	26.27	4.58
**6e**	H	4-CN-Bn	>100	>100	48.45	59.13	4.82
**6f**	NO_2_	4-CN-Bn	>100	>100	64.33	50.45	6.49
**AAZ**	–	–	0.25	0.012	0.011	0.0025	0.025

^a^ Mean from three different assays, by a stopped-flow technique (errors were in the range of ±5–10% of the reported values).

The following structure–activity relationship (SAR) can be observed regarding the inhibition data of Table 1:

- The ubiquitous isoform hCA I was not inhibited at all by the newly developed hydantoin derivatives. On the other hand, the second widely expressed isoform hCA II was poorly inhibited by three derivatives (**3a**, **3b**, and **5**) with inhibition constants in the range of 6.94–58.58 μM, all of which possess the free –NH/K group.
- The unique secreted isoform hCA VI was on the other hand moderately to poorly inhibited by the phthalimide–hydantoin hybrids reported here, with *K*_I_s in the submicromolar to micromolar ranges, more precisely 0.405–64.33 μM (Table 1). Again, the best inhibitors were salts **3a** and **3b**; albeit, being 37- and 64-fold, respectively, inferior to AAZ whose *K*_I_ value is 0.011 μM. High inhibitory activity of these salts indicated that this class of compounds bind to the CA Zn ion via the imidic nitrogen of the hydantoin nucleus.
- The other cytosolic isoform, hCA VII, was affected efficiently by the newly developed compounds, as compounds **3a** and **3b** showed potent activity inhibition constants in the sub-micromolar ranges (0.075 and 0.163 μM, respectively). However, compounds **5** and **6a–f** showed weak to moderate activity against hCA VII (*K*_I_s = 13.88–59.13 μM). It should be mentioned that the electronic effects of substituents on the phenyl ring periphery of *N*-benzyl substituted derivatives (**6a**–**f**) almost did not influence their inhibitory capability.
- The extracellular, tumour-associated isoform hCA IX was moderately to poorly inhibited by the tested compounds with inhibition constants in the range of 0.331–6.49 μM. Like all other isoforms, in this case, potassium 3-(1,3-dioxoisoindolin-2-yl)-2,5-dioxoimidazolidin-1-ide **3a** demonstrated the best activity, with a *K*_I_ value of 0.331 μM but was still 13-fold less potent than AAZ (*K*_I_ of 0.025 μM).

### Computational chemistry studies

**Figure 3.**
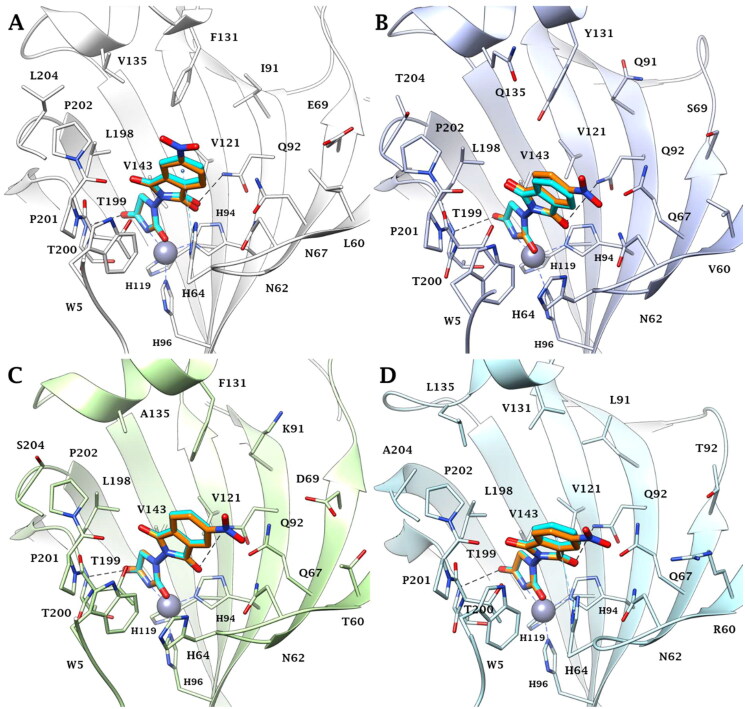
Predicted binding mode of ligands **3a** (cyan) and **3b** (orange) within the human: (A) CA II, (B) CA VI, (C) CA VII, and (D) CA IX active site. H-bonds and π–π stacking interactions are represented as black and cyan dashed lines, respectively.

Predictions on the binding mode and studies on the ligand/target interactions for the most active compounds **3a**, **3b**, and **5** against the isoforms I, II, VI, VII, and IX of human carbonic anhydrases (Table 1) were carried out by docking calculations. Solutions were found for all the isoenzymes studied, except for hCA I; in this case, residues H67, F91, and H200 prevent ligand binding and the inhibition profiles are actually very poor in this isoform. In the other isoforms, the hydantoin scaffold deeply bound to the zinc ion with the deprotonated imidic nitrogen atom (CON^−^CO) for ligands **3a** and **3b**, according to the literature ^14^ (Figure 3). The binding of ligands completed the tetrahedral coordination sphere of the metal and the overall stabilisation is reinforced by an H-bond between the C=O in position 4 of the hydantoin scaffold and the backbone NH of T199 and by van der Waals (vdW) contacts occurring between the heterocycle and the residues H94, H96, H119, L198, T200, and W209.

The isoindoline-1,3-dione scaffold is able to engage π–π stacking interactions with the proton shuttle residue H64 (Figure 3(A,D)) and the C=O group is in H-bond distance with the side chain NH_2_ of the conserved Q92 (Figure 3(A–D)) in the active sites of all investigated isozymes.

Because of the mutation N67/Q97 (hCA II/hCA VI, VII, and IX), the improved ligand/isoforms matching makes the investigated compounds selective for hCA VI, VII, and IX compared to the hCA II (Figure 3). Other mutations, such as Y131/F131 (hCA VI/hCA VII) or Y131/V131 (hCA VII/hCA IX), may lead to decreased inhibitory activity of ligands **3a** and **3b** in hCA VI due to the polar character of Y131 and to steric hindrance associated to its phenolic OH (Figure 3(B,C)).

Instead, the presence of V131 in hCA IX active site decreases the ability of the target to stabilise the ligands binding mode by vdW interactions (Figure 3(C,D)).

**Figure 4.**
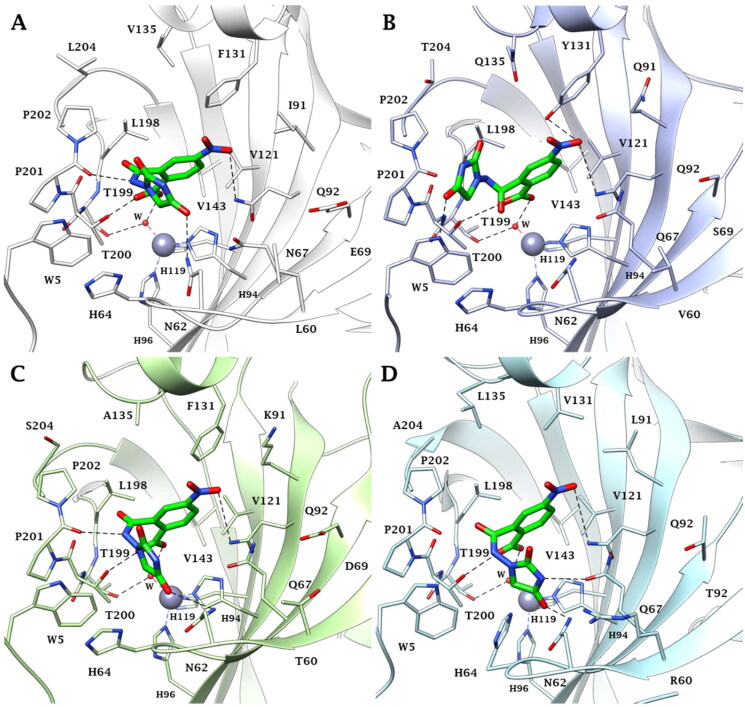
Predicted binding mode of ligand **5** (green) within the human: (A) CA II, (B) CA VI, (C) CA VII, and (D) CA IX active site. H-bonds and π–π stacking interactions are represented as black and cyan dashed lines, respectively.

Several literature data^4^ reporting the CA inhibition mechanism of ligands bearing the carboxylic group support the hypothesis that the binding of compound **5** to the metal ion occurs via a Zn-bonded water molecule (Figure 4). The carboxylate group interacts with the oxygen HOH lone-pairs forming, in addition, a hydrogen bond with the side chain OH of the conserved T200. Complete the stabilisation pattern of the H-bond occurring between the water molecule and the side chain OH of T199.

In hCA II and hCA VII active sites, the amidic NH, imidic C=O and NO_2_ group are in H-bond distance with the backbone C=O of P201, and with the N62 and Q92 side chain NH_2_, respectively (Figure 4(A,C)). Because of the mutations N67/Q67 and I91/K91 (hCA II/hCA VII) and the resulting change in the network of hydrophobic contacts or steric hindrance, the inhibitory profile of **5** is modulated differently in these isoforms.

In the case of the secreted-isoform hCA VI, while the imidic C=O engages an H-bond with the indolic NH of conserved W5, the NO_2_ group is in H-bond distance with the side chain NH_2_ of Q92 and with the side chain OH of the peculiar Y131 (Figure 4(B)). It is likely that the specificity of this latter interaction plays a role in the better *K*_I_ value of compound **5** for hCA VI over hCA II and VII.

In conclusion, the binding of compound **5** within the tumour-associated hCA IX is favoured by residues Q67 and V131, peculiar to this isoform. In fact, due to the lower bulky nature of V131, the compound is able to accommodate well within the binding cavity and to establish direct interactions with Q67. This could explain the better hCA IX inhibition profile of derivative **5** over the hCA II, VI, and VII isoforms (Figure 4(D)).

## Experimental

### Chemistry methods

All the chemicals used were of analytical grade purity. Thin-layer chromatography was performed on silica gel, spots were visualised with UV light (254 and 365 nm).^1^H and ^13^C NMR spectra were registered in DMSO-*d*_6_ using Bruker 500 MHz instrument (Billerica, MA). NMR multiplicities are abbreviated as follows: s = singlet, d = doublet, t = triplet, q = quartette, m = multiplet, and br = broad signal. Chemical shifts (*δ*) are given in parts per million (ppm) and are referenced to TMS (^1^H,^13^C). High-resolution mass spectra (HRMS) were recorded on a mass spectrometer with a Q-TOF micro mass analyser using the ESI technique.

### Synthesis

#### General procedure (A) for preparation of ethyl 1,3-dioxoisoindoline-2-carboxylates (2)

To an ice-cooled solution of the appropriate isoindoline-1,3-dione (50 mmol, 1.0 equiv.) and Et_3_N (60 mmol, 1.2 equiv.) in dry DMF (100 ml) was dropwise added ethyl chloroformate (50 mmol, 1.0 equiv.). After 1 h of stirring at 0 °C, the reaction flask was allowed to warm to 20 °C and the mixture was stirred at the indicated temperature for 4 h. Then, the mixture was quenched with water (200 ml) and the formed precipitate was filtered, washed with water (500 ml) and Et_2_O (50 ml) and dried under vacuum to give the final desired products as off-white solids.

##### Ethyl 1,3-dioxoisoindoline-2-carboxylate (2a)

Following the general procedure A, **2a** was obtained as a white solid. (6.80 g, 48% yield). ^1^H NMR (500 MHz, DMSO-*d*_6_) *δ* = 1.36 (t, 3H, *J* = 7.1 Hz), 4.40 (q, 2H, *J* = 7.1 Hz), 7.95–8.00 (m, 4H) ppm. ^13^C NMR (125 MHz, DMSO-*d*_6_) *δ* = 14.9, 64.2, 124.9, 131.8, 136.4, 149.0, and 164.6 ppm.


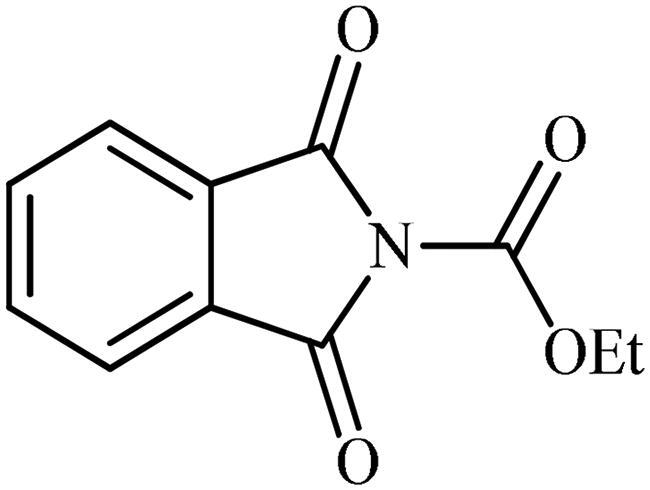


##### Ethyl 5-nitro-1,3-dioxoisoindoline-2-carboxylate (2b)

Following the general procedure A, **2b** was obtained as a white solid (9.56 g, 72% yield). ^1^H NMR (500 MHz, DMSO-*d*_6_) *δ* = 1.37 (t, 3H, *J* = 7.0 Hz), 4.44 (q, 2H, *J* = 7.0 Hz), 8.24 (d, 1H, *J* = 8.2 Hz), 8.60 (s, 1H), 8.71 (d, 1H, *J* = 8.2 Hz) ppm. ^13^C NMR (125 MHz, DMSO-*d*_6_) *δ* = 14.9, 64.5, 119.7, 126.5, 131.2, 133.4, 136.5, 148.6, 152.8, 163.0, and 163.2 ppm.


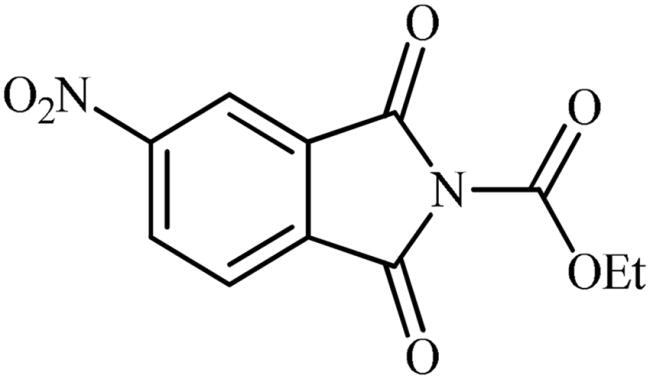


#### General procedure (B) for preparation of potassium 3-(1,3-dioxoisoindolin-2-yl)-2,5-dioxoimidazolidin-1-ides (3)

The appropriate ethyl 1,3-dioxoisoindoline-2-carboxylate (10 mmol, 1.0 equiv.) and 1-aminohydantoin hydrochloride (10 mmol, 1.0 equiv.) were added to NMP (40 ml) at room temperature. Then, K_2_CO_3_ (20 mmol, 2.0 equiv.) was slowly added to the above reaction mixture and the reaction mixture was stirred for 20 h at 100 °C. After completion of the reaction, the mixture was quenched with *^t^*BuOMe (400 ml) and the formed precipitate was filtered, washed with *^t^*BuOMe (100 ml) and MeOH (200 ml) and dried under vacuum to give the final desired products.

##### Potassium 3-(1,3-dioxoisoindolin-2-yl)-2,5-dioxoimidazolidin-1-ide (3a)

Following the general procedure B, **3a** was obtained as a white solid (2.35 g, 83% yield). Notably, 0.74 g of KCl was also participated with the product. ^1^H NMR (500 MHz, D_2_O) *δ* = 4.06 (s, 2H), 7.45–7.53 (m, 3H), 7.59–7.61 (m, 1H) ppm. ^13^C NMR (125 MHz, D_2_O) *δ* = 53.8, 127.5, 128.3, 129.6, 131.1, 131.7, 137.9, 172.3, 173.2, 175.2, and 187.8 ppm. HRMS (ESI) [M + H]^+^: *m*/*z* calcd. for (C_11_H_8_N_3_O_4_) 246.0515. Found 246.0513.


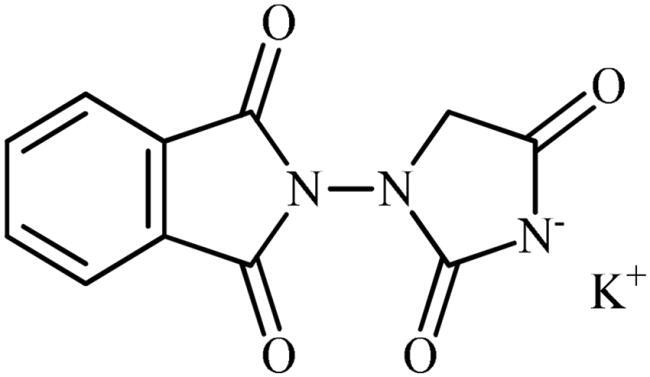


##### Potassium 3-(5-nitro-1,3-dioxoisoindolin-2-yl)-2,5-dioxoimidazolidin-1-ide (3b)

Following the general procedure B, **3b** was obtained as a white solid. (1.59 g, 48% yield). Notably, 0.74 g of KCl was also participated with the product. ^1^H NMR (500 MHz, D_2_O) *δ* = 4.16 (s, 2H), 7.73–7.78 (m, 1H), 8.29–8.35 (m, 1H), 8.45 and 8.46 (s, 1H) ppm. ^13^C NMR (125 MHz, D_2_O) *δ* = 153.1, 53.2, 123.0, 123.7, 124.7, 126.2, 129.0, 129.5, 132.4, 138.1, 139.2, 144.3, 147.4, 148.7, 167.3, 167.4, 169.7, 170.9, 171.8, 173.0, 181.7, and 181.8 ppm. HRMS (ESI) [M + Na]^+^: *m*/*z* calcd for (C_11_H_6_N_4_NaO_6_) 313.0185. Found 315.0352.


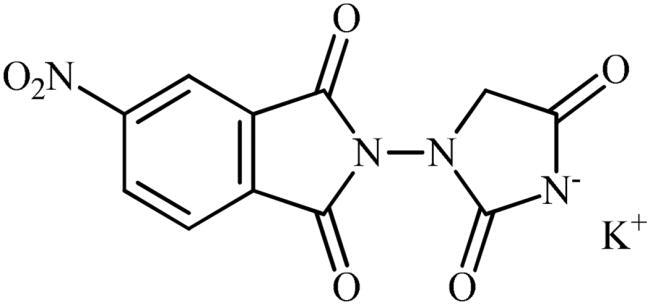


#### 2-(2,4-dioxoimidazolidin-1-yl)isoindoline-1,3-dione (4)

To a solution of potassium 3-(1,3-dioxoisoindolin-2-yl)-2,5-dioxoimidazolidin-1-ide (**3a**) (300 mg, 1.06 mmol) in H_2_O (3 ml) under stirring, HCO_2_H (0.3 ml) was slowly added and the mixture was heated at 70 °C for 2 h. After cooling to room temperature, the formed precipitate was filtered, washed with water (30 ml) and Et_2_O (10 ml) and dried under vacuum to afford **4** (140 mg, 54%) as a white powder.

^1^H NMR (500 MHz, DMSO-*d*_6_) *δ* = 4.34 (s, 2H), 7.99–8.02 (m, 2H), 8.03–7.06 (m, 2H), 11.81 (s, 1H) ppm. ^13^C NMR (125 MHz, DMSO-*d*_6_) *δ* = 54.4, 124.9, 130.2, 136.5, 157.5, 165.3, and 170.4 ppm. HRMS (ESI) [M + H]^+^: *m*/*z* calcd for (C_11_H_8_N_3_O_4_) 246.0515. Found 246.0517.


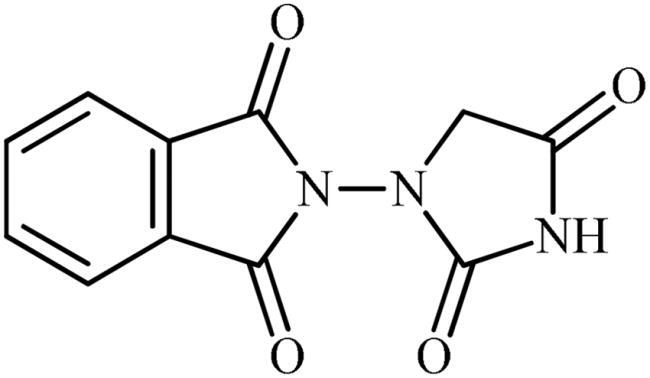


#### 2-((2,4-Dioxoimidazolidin-1-yl)carbamoyl)-4-nitrobenzoic acid (5)

To a solution of potassium 3-(5-nitro-1,3-dioxoisoindolin-2-yl)-2,5-dioxoimidazolidin-1-ide (**3b**) (300 mg, 0.91 mmol) in H_2_O (3 ml) under stirring, HCO_2_H (0.3 ml) was slowly added and the mixture was heated at 50 °C for 30 min. After cooling to room temperature, the formed precipitate was filtered, washed with water (30 ml) and Et_2_O (10 ml) and dried under vacuum to afford **5** (135 mg, 48%) as a white powder.

^1^H NMR (500 MHz, DMSO-*d*_6_) *δ* = 4.17 (s, 2H), 7.76 (s, 1H), 8.58 (d, 2H, *J* = 39.6 Hz), 10.87 (s, 1H), 11.37 (s, 1H), 14.06 (s, 1H) ppm. ^13^C NMR (125 MHz, DMSO-*d*_6_) *δ* = 52.5, 125.5, 127.9, 130.8, 132.6, 141.8, 149.1, 158.5, 166.1, 167.8, and 171.0 ppm. HRMS (ESI) [M + Na]^+^: *m*/*z* calcd for (C_11_H_8_N_4_NaO_7_) 331.0291. Found 331.0294.


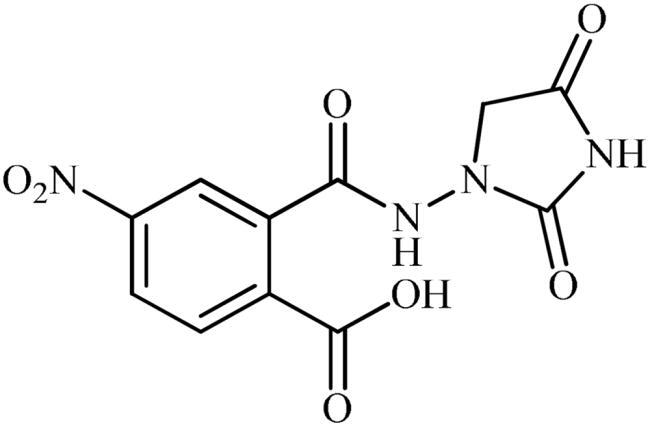


#### General procedure (C) for preparation of 2-(3-benzyl-2,4-dioxoimidazolidin-1-yl)isoindoline-1,3-diones (6a–f)

To a suspension of appropriate potassium 3-(1,3-dioxoisoindolin-2-yl)-2,5-dioxoimidazolidin-1-ide (1.0 mmol, 1.0 equiv.) in DMSO (4 ml) was added the appropriate benzyl bromide (1.5 mmol, 1.5 equiv.) and the mixture was stirred at 100 °C for 5 h. The mixture was cooled to room temperature and water (80 ml) and Et_2_O (10 ml) were added to the mixture and the solution was vigorously stirred for 30 min. Subsequently, the solids formed in the organic phase were filtered and washed with water and Et_2_O to afford the desired 2-(3-benzyl-2,4-dioxoimidazolidin-1-yl)isoindoline-1,3-dione derivatives. Notably, in the case of **6b**, after the reaction was completed, the mixture was poured into ethyl acetate, washed with brine, extracted with ethyl acetate, dried over Na_2_SO_4_, filtered, and dried under vacuum, and finally the oily residue was crystallised in *^i^*PrOH.

##### 2-(3-Benzyl-2,4-dioxoimidazolidin-1-yl)isoindoline-1,3-dione (6a)

Following the general procedure C, **6a** was obtained as a white solid (57 mg, 17% yield). ^1^H NMR (500 MHz, DMSO-*d*_6_) *δ* = 4.48 (s, 2H), 4.74 (s, 2H), 7.34–7.44 (m, 5H), and 7.98–8.08 (m, 4H) ppm. ^13^C NMR (125 MHz, DMSO-*d*_6_) *δ* = 43.0, 52.6, 125.0, 128.4, 128.6, 129.6, 130.2, 136.5, 136.7, 157.1, 165.2, and 168.9 ppm. HRMS (ESI) [M + H]^+^: *m*/*z* calcd for (C_18_H_14_N_3_O_4_) 336.0984. Found 336.0998.


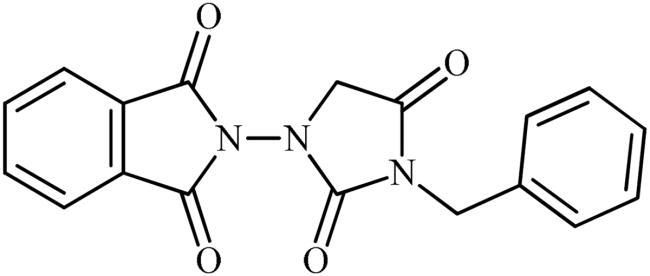


##### 2-(3-Benzyl-2,4-dioxoimidazolidin-1-yl)-5-nitroisoindoline-1,3-dione (6b)

Following the general procedure C, **6b** was obtained as a white solid (103 mg, 27% yield). ^1^H NMR (500 MHz, DMSO-*d*_6_) *δ* = 4.49 (s, 2H), 4.75 (s, 2H), 7.34–7.38 (m, 3H), 7.40–7.44 (m, 2H), 8.31 (d, 1H, *J* = 7.9 Hz), 8.69 (s, 1H), and 8.76 (d, 2H, *J* = 7.9 Hz) ppm. ^13^C NMR (125 MHz, DMSO-*d*_6_) *δ* = 43.0, 52.5, 119.9, 126.6, 128.4, 128.7, 129.6, 131.5, 131.6, 134.8, 136.6, 152.9, 156.9, 163.4, 163.7, and 168.8 ppm. HRMS (ESI) [M + H]^+^: *m*/*z* calcd for (C_18_H_13_N_4_O_6_) 381.0835. Found 381.0836.


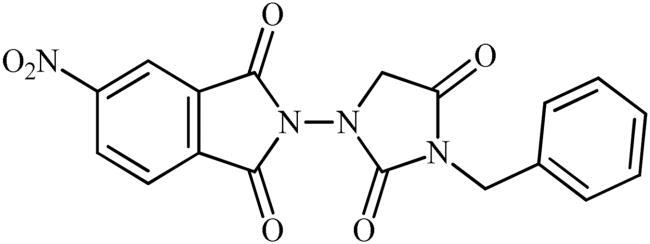


##### 2-(3-(4-Nitrobenzyl)-2,4-dioxoimidazolidin-1-yl)isoindoline-1,3-dione (6c)

Following the general procedure C, **6c** was obtained as a yellow solid (61 mg, 16% yield). ^1^H NMR (500 MHz, DMSO-*d*_6_) *δ* = 4.51 (s, 2H), 4.90 (s, 2H), 7.64 (d, 2H, *J* = 4.7 Hz), 7.99–8.08 (m, 4H), and 8.29 (d, 2H, *J* = 4.7 Hz) ppm. ^13^C NMR (125 MHz, DMSO-*d*_6_) *δ* = 42.4, 52.8, 124.8, 125.0, 129.6, 130.2, 136.6, 144.2, 148.0, 156.9, 165.1, and 169.0 ppm. HRMS (ESI) [M + H]^+^: *m*/*z* calcd for (C_18_H_13_N_4_O_6_) 381.0835. Found 381.0816.


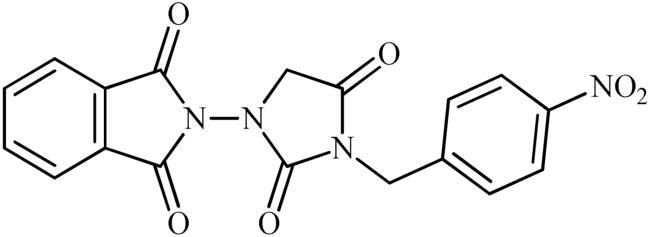


##### 5-Nitro-2-(3-(4-nitrobenzyl)-2,4-dioxoimidazolidin-1-yl)isoindoline-1,3-dione (6d)

Following the general procedure C, **6d** was obtained as a white solid (178 mg, 42% yield). ^1^H NMR (500 MHz, DMSO-*d*_6_) *δ* = 4.52 (s, 2H), 4.90 (s, 2H), 7.64 (d, 2H, *J* = 7.7 Hz), 8.29 (d, 2H, *J* = 7.7 Hz), 8.31 (d, 1H, *J* = 7.5 Hz), 8.69 (s, 1H), and 8.76 (d, 1H, *J* = 7.5 Hz) ppm. ^13^C NMR (125 MHz, DMSO-*d*_6_) *δ* = 42.5, 52.6, 119,9, 124.8, 126.6, 129.6, 131.5, 131.6, 134.8, 144.1, 148.0, 152.9, 156.7, 163.4, 163.6, and 168.9 ppm. HRMS (ESI) [M + H]^+^: *m*/*z* calcd for (C_18_H_12_N_5_O_8_) 426.0686. Found 426.0691.


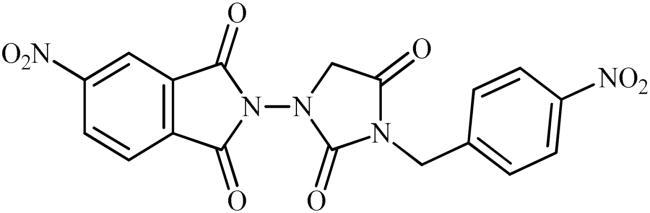


##### 4-((3-(1,3-Dioxoisoindolin-2-yl)-2,5-dioxoimidazolidin-1-yl)methyl)benzonitrile (6e)

Following the general procedure C, **6e** was obtained as a white solid (68 mg, 19% yield). ^1^H NMR (500 MHz, DMSO-*d*_6_) *δ* = 4.50 (s, 2H), 4.84 (s, 2H), 7.55 (d, 2H, *J* = 7.3 Hz), 7.91 (d, 2H, *J* = 7.3 Hz), and 7.99–8.08 (m, 4H) ppm. ^13^C NMR (125 MHz, DMSO-*d*_6_) *δ* = 42.6, 52.7, 111.5, 119.5, 125.0, 129.1, 130.2, 133.6, 136.5, 142.2, 156.9, 165.1, and 169.0 ppm. HRMS (ESI) [M + H]^+^: *m*/*z* calcd for (C_19_H_13_N_4_O_4_) 361.0937. Found 361.0935.


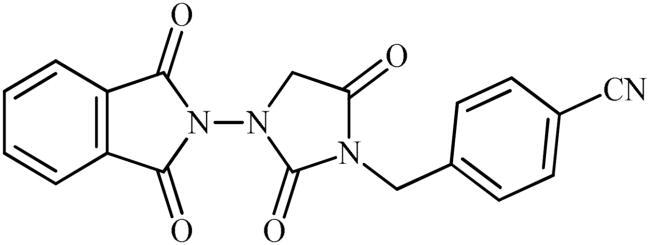


##### 4-((3-(5-Nitro-1,3-dioxoisoindolin-2-yl)-2,5-dioxoimidazolidin-1-yl)methyl)benzonitrile (6f)

Following the general procedure C, **6f** was obtained as a white solid (259 mg, 64% yield). ^1^H NMR (500 MHz, DMSO-*d*_6_) *δ* = 4.52 (s, 2H), 4.85 (s, 2H), 7.55 (d, 2H, *J* = 7.3 Hz), 7.92 (d, 2H, *J* = 7.3 Hz), 8.31 (d, 2H, *J* = 7.6 Hz), 8.69 (s, 1H), and 8.76 (d, 2H, *J* = 7.6 Hz) ppm. ^13^C NMR (125 MHz, DMSO-*d*_6_) *δ* = 42.7, 52.6, 111.6, 119.6, 119.9, 126.6, 129.2, 131.5, 131.6, 133.6, 134.9, 142.2, 152.9, 156.8, 163.4, 163.7, and 168.9 ppm. HRMS (ESI) [M + H]^+^: *m*/*z* calcd for (C_19_H_12_N_5_O_6_) 406.0788. Found 406.0785.


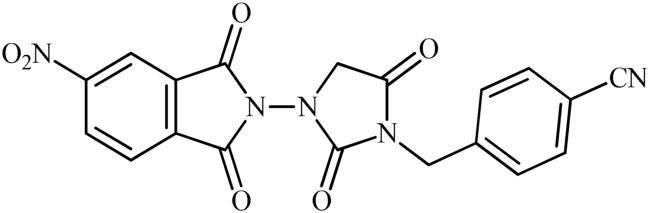


### CA inhibitory assay

An applied photophysics stopped-flow instrument was employed to determine the CA catalysed CO_2_ hydration activity,^16^ as described earlier by our group^17^^,^^18^. The tested CA enzymes were in-house produced recombinant proteins, that their preparation procedures have previously been reported^18^^,^^19^. Briefly, cDNAs were expressed in *Escherichia coli* strain BL21 (DE3) from the plasmids pACA/hCA I and pACA/hCA II (for hCA I and II) and of the catalytic domains of hCA IX and XII.^14^ The constructs were inserted in the pCAL-n-FLAG vector and then cloned and expressed in *Escherichia coli* strain BL21-GOLD(DE3). The bacterial cells were lysed and homogenated in a buffered solution (pH 8) of 4 M urea and 2% Triton X-100. The homogenates thus obtained were centrifuged (11 000 × *g*) for removing soluble and membrane associated proteins and cellular debris. The pellets were washed by repeated homogenisation and centrifugation in water, in order to remove the remaining urea and Triton X-100. Enzymes were thereafter purified by column chromatography on sulphonamide based columns.^4^ The amount of proteins was determined by spectrophotometric measurements and their activities by stopped-flow measurements, with CO_2_ as substrate.^16^

### Docking studies

The crystal structures CA II (PDB 3K34)^18^, CA VI (alphafold model) ^20^ CA VII (PDB 6H38) ^21^, and CA IX (PDB 5FL4)^22^ were downloaded by Protein Data Bank (RCSB.org)^23^ and prepared using the Protein Preparation module implemented in Maestro Schrödinger suite,^24^ assigning bond orders, adding hydrogens, deleting water molecules, and optimising H-bonding networks. Finally, energy minimisation with a root mean square deviation (RMSD) value of 0.30 was applied using an optimised potential for liquid simulation (OPLS4) force field.^25^ The 3D ligand structures were prepared by Maestro^23^^b^ and evaluated for their ionisation states at pH 7.3 ± 1.0 with Epik^23^^c^. The conjugate gradient method in Macromodel^24^ was used for energy minimisation (maximum iteration number: 2500; convergence criterion: 0.05 kcal/mol/Å^2^). Grids for docking were centred in the centroid of the complexed ligand. Docking studies were carried out with the program Glide^24^ using the standard precision (SP) mode. Figures were generated with Maestro and Chimera^24^^,^^26^.

## Conclusions

We have synthesised a small series of hitherto unknown phthalimide–hydantoin hybrids and screened them against five human CA isoforms: the cytosolic isoforms hCA I, II, and VII as well as the unique secreted isoform hCA VI and the trans-membrane tumour-associated isoform hCA IX. All compounds showed potent inhibitory activity against hCA II, VI, VII, and IX whereas they did not display any inhibitory activity towards ubiquitous cytosolic isoform hCA I. Among them, ionic compounds **3a** and **3b** exhibited superior inhibitory activity, followed by derivative **5**, and their binding mode was proposed by *in silico* studies. The SAR indicated that blocking the N3-position of the hydantoin core resulted in a strong decrease in activity. Interestingly, hydantoin salt **3a** (an NH-free hydantoin derivative) exhibited much better inhibitory against tumour-associated CA IX compared to all newly developed Furagin derivatives (*K*_I_s = 0.35–7.3 μM, average: 2.84 μM). Importantly, this compound also showed excellent selectivity towards CA IX over the dominant cytosolic isoforms CA I and II, far better than the reference drug, AAZ. Therefore, it may be considered as a promising starting point for the development of novel anti-cancer agents.

## Data Availability

Additional data may be requested from the authors.
